# *Gastrodia elata* Blume Polysaccharides Attenuate Vincristine-Evoked Neuropathic Pain through the Inhibition of Neuroinflammation

**DOI:** 10.1155/2021/9965081

**Published:** 2021-07-27

**Authors:** Hengtao Xie, Yingying Chen, Wei Wu, Xiaobo Feng, Kairong Du

**Affiliations:** Department of Anesthesiology, Zhongnan Hospital of Wuhan University, Wuhan, 430071 Hubei, China

## Abstract

Vincristine (Vin) is a well-known antitumor agent that frequently evokes neuropathic pain and decreases the quality of life of patients. Polysaccharides (GBP) extracted from *Gastrodia elata* Blume have been demonstrated to possess anti-inflammatory and neuroprotective effects *in vivo*; however, the effects of GBP on Vin-induced neuropathic pain remain unknown. The present study is aimed at exploring the alleviative potential of GBP against chemotherapy-evoked peripheral neuropathy to better understand and extend its pharmacological application. Vin was administered intraperitoneally to evoke neuropathic pain. GBP was orally administered for 21 days. The mechanical allodynia and thermal hyperalgesia were assessed using the Von Frey test and hot-plate test. Histopathological changes were assessed by hematoxylin and eosin staining. ELISA kits were used to measure the levels of inflammatory cytokines in the sciatic nerve, spinal cord, and dorsal root ganglion (DRG). qRT-PCR was employed to examine the expression of inflammatory cytokines and Sirtuin1 (SIRT1) in the sciatic nerve, spinal cord, and DRG. Our findings revealed that GBP treatment enhanced the paw withdrawal latency and paw withdrawal threshold and restored Vin-induced sciatic nerve damage in rats. GBP also attenuated the Vin-induced increase of proinflammatory cytokine levels, including IL-6, IL-8, TNF-*α*, IL-1*β*, and NF-*κ*B. On the molecular level, treatment with GBP downregulated the mRNA levels of IL-6, IL-8, TNF-*α*, and IL-1*β* in the sciatic nerve, spinal cord, and DRG. Meanwhile, GBP increased SIRT1 activity and mRNA expression levels. Our data indicated that GBP exerted a potential protective effect against chemotherapy-induced neuropathic pain which might be mediated via the inhibition of neuroinflammation.

## 1. Introduction

Neuropathic pain is one of the most distressing and common symptoms caused by an impairment affecting the central nerve or peripheral nerve system. It is characterized by sensory deformities, including spontaneous pain, hyperalgesia, and allodynia, which not only cause mental disorders and depression but also affect the sleep quality of patients [1]. Vincristine (Vin) is a prescribed chemotherapeutic drug for the treatment of various tumors, such as brain tumors, breast cancer, Hodgkin's lymphoma, and acute lymphoblastic leukemia [2]. Unfortunately, the long-term use of Vin could induce severe peripheral neuropathic pain, which is a dose-limiting neurotoxic effect [3]. Neuropathic pain remains one of the most challenging diseases, and the current therapies for chemotherapy-induced neuropathic pain are limited, partly due to a poor understanding of the underlying mechanisms of action.

Accumulating evidence indicated that inflammation responses are considered to be involved in the pathogenesis of neuropathic pain [4]. A previous study has demonstrated that chemotherapy-induced neuropathy is associated with the induction of proinflammation mediators (such as IL-1*β* and TNF-*α*) in the dorsal root ganglion (DRG), which may ultimately cause the initiation and maintenance of persistent neuropathy [5]. Therefore, the inhibition of proinflammatory cytokine action in the central nervous system is a potential treatment strategy for peripheral neuropathy. It has been reported that Sirtuin1 (SIRT1) plays a vital role in the progression of diabetic neuropathic pain via the regulation of synaptic plasticity at the spinal dorsal horn [6]. Moreover, a previous study indicated that microRNA-448 promoted the progression of neuropathic pain through the regulation of neuroinflammation via targeting SIRT1 [7]. Thus, SIRT1 may be considered a novel target for peripheral neuropathy.

A rhizome of *Gastrodia elata* Blume (*G. elata*) is a precious traditional herbal medicine that has been used for thousands of years for the treatment of limb numbness, epilepsy, dizziness, and headaches because of its anti-inflammatory, nootropic, and analgesic effects [8, 9]. Numerous active ingredients have been identified, and their pharmacological activities have been investigated, including phenolic compounds, parishin, gastrodin, and polysaccharides [10, 11]. *G. elata* polysaccharides (GBP), as the primary active constituents of *G. elata* root, were demonstrated to have several pharmacological activities, such as improving lipid metabolism [12], lowering hypertension [12], and being neuroprotective [13]. However, there is little research about the analgesic effect of GBP in a chemotherapy-induced neuropathy model. Besides, the activity of GBP on inflammatory pain has never been explored.

Based on the neuroprotective effects of GBP in the previous studies, we hypothesized that GBP could alleviate neuropathic pain symptoms. Thus, the present study is aimed at exploring the neuroprotective role of GBP using the Vin-induced neuropathic pain model.

## 2. Material and Methods

### 2.1. Material

*G. elata* root was obtained from Shiyan city (Hubei, China) and authenticated by Professor Hengtao Xie of Zhongnan Hospital of Wuhan University. Vin, bovine serum albumin, galacturonic acid, and D-glucose were purchased from Sigma-Aldrich (St. Louis, USA). All other reagents used were of analytical grade.

### 2.2. Preparation of GBP

The root of *G. elata* was ground and passed using a 100-mesh sieve. About 1000 g of *G. elata* powder was extracted two times with 10 L 75% ethanol for 24 h at room temperature. Subsequently, the ethanol solvent was filtered and the residue was dried for 24 h at 60°C. Then, the *G. elata* residue was extracted two times with 5000 mL water at 80°C (each time is 45 min). The aqueous extraction solution was filtered again and concentrated at 60°C under reduced pressure conditions. Then, the condensate was precipitated with 4 volumes of 80% ethanol for 24 h at 3°C and centrifuged at 4000 g for 15 min to obtain the precipitate. Finally, GBP was obtained by lyophilization [14]. The total polysaccharide content was measured using the phenol sulfuric method with D-glucose as the standard.

### 2.3. Measurement of Molecular Weight and Homogeneity

Gel permeation chromatography combined with a multiangle laser light scattering detector and refractive index detector (GPC-MALLS-RI) was used to measure the molecular weight and homogeneity of GBP. A Waters 2695 HPLC System equipped with a multiangle laser light scattering detector (Wyatt Technology Co), a 2414 refractive index detector (RI), and a Shodex-OHpak SB-805 HQ column (8.0 mm × 300 mm) was used. The column was eluted with a NaCl solution (5 mM) at a flow rate of 0.7 mL/min, and the temperature of the column was maintained at 35°C. The GBP was dissolved with 5 mM of NaCl solution (2 mg/mL), and the injection volume was 50 *μ*L.

### 2.4. Monosaccharide Composition Analysis of GBP

An HPLC-RI analytical method was performed to measure the monosaccharide composition of GBP. Briefly, the GBP sample (10 mg) was hydrolyzed with 1000 *μ*L of trifluoroacetic acid (2 mol/L) at 120°C for 2 h. The reaction solution was cooled after hydrolysis and then evaporated under a vacuum to dry. The dried sample was dissolved in the mobile phase. An ODS-NH2 column (4.6 × 150 mm, 5 *μ*m; ZHONGPU SCIENCE, China) was used to analyze the monosaccharide composition, and the analysis conditions were as follows: a RI refractive index detector (Waters, USA), injection volume 10 *μ*L, column temperature 40°C, and 80% acetonitrile (aq.) for isocratic elution.

## 3. Animal Experiments

### 3.1. Animals

Adult male Sprague-Dawley rats (180-220 g) were purchased from Hubei province Laboratory Animal Center. The housing conditions of the animals were as follows: a 12/12 h light/dark cycle in 55% ± 5% humidity at temperature 20°C ± 2°C, with water and food *ad libitum*. The animal experiment was in the accordance with the *Guide for the Care and Use of Laboratory Animals* and approved by the Ethics Committee of the Zhongnan Hospital of Wuhan University.

### 3.2. Animal Model of Neuropathic Pain and Drug Administration

**Figure 1:**
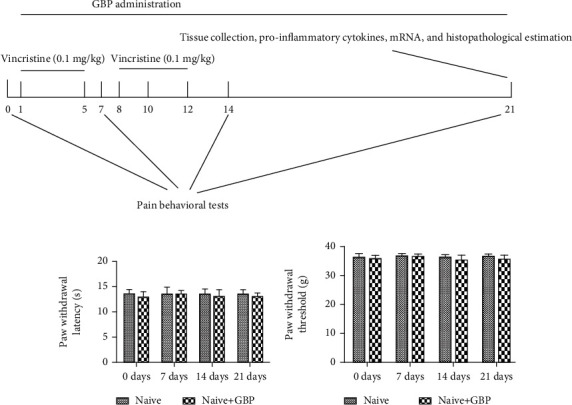
Detailed experimental protocol representing the treatment of GBP (a). Effects of GBP on the paw withdrawal latency (b) and paw withdrawal threshold (c) in normal rats. Data were expressed as the mean ± SD (*n* = 8 for each group).

After acclimatization of one week, rats were randomly divided into four groups as follows (each group consists of eight rats): naive group, control rats received 5% DMSO; naive+GBP group, control rats received GBP (20 mg/kg); vincristine (Vin) group, rats received 5% DMSO and Vin injection; and Vin+GBP group, rats received GBP (20 mg/kg) and vincristine injection. Except for the naive group and naive+GBP group, rats in the other groups were administrated with Vin (0.1 mg/kg) intraperitoneally after 2 h of GBP administration in a two-five-day cycle with a two-day pause in between (from day 1 to 5 and from day 8 to 12) [15]. The dose of GBP was based on the previous research and our preliminary experiments [16]. Rats received 5% DMSO vehicle or GBP (dissolved in 5% DMSO) via oral gavage once a day for 21 consecutive days. The detailed experimental schedule is shown in Figure 1(a).

### 3.3. Pain Behavioral Assessment

Pain behavioral assessments were carried out from 9:00 a.m. to 5:00 p.m. 1 h after GBP administration on the 0^th^, 7^th^, 14^th^, and 21^st^ day. The paw withdrawal latency was measured using a hot plate test to evaluate the thermal hyperalgesia. Briefly, the rats were individually placed on a hot plate analgesia meter (BIO-CHP, Bioseb, France) with the temperature heated to 52 ± 1°C. The paw latency reaction times, namely, the paw jumping, flinching, and/or licking, were taken as an index of the pain threshold. A cut-off time of 20 s was used to avoid the paw damage.

The paw withdrawal threshold was measured by Von Frey filaments to evaluate the mechanical allodynia. Briefly, the rats were kept in a plastic cage and acclimatized for 30 min before the measurement. A series of Von Frey filaments were used (ranging from 0 to 50 g) perpendicular to the plantar surface of the hind paw. In the case of the paw withdrawal, the next smaller filament was used; the next larger filament was used if no paw withdrawal was elicited. The paw withdrawal threshold was defined as the minimum force required to cause a withdrawal reaction from the paw. Each Von Frey test was repeated three times at 5 min intervals, and the withdrawal threshold was defined as the average forge.

### 3.4. Pathological Examination

For this experiment, after completion of the pain tests on day 21, the rats were anesthetized with pentobarbital sodium (50 mg/kg), and the sciatic nerves were collected after the rats were perfused transcardially with 0.9% saline and with 4% paraformaldehyde. Then, samples of the sciatic nerve were fixed in 10% formalin overnight at 4°C. The samples were then embedded in paraffin and sliced (approximately 4 *μ*m). The paraffin sections were stained with the hematoxylin and eosin staining reagent and viewed under a light microscope (DMi8, Leica Microscope Ltd., Wetzlar, Germany).

### 3.5. Enzyme-Linked Immunosorbent Assay (ELISA)

After completion of the pain behavioral tests on day 21, the rats were anesthetized with pentobarbital sodium (50 mg/kg). Sciatic nerves, ipsilateral lumbar spinal cord dorsal horn (L4-L5), and the corresponding dorsal root ganglion (DRG) tissues were rapidly collected and homogenated with ice normal saline (1/9, *m*/*v*) by using a homogenizer and centrifuged at 12,000 g for 15 min at 4°C, and the supernatant of the homogenate was collected and used to measure the IL-6, IL-8, TNF-*α*, IL-1*β*, and NF-*κ*B levels according to the manufacturer's instructions from the commercial ELISA kits (Nanjing Jiancheng Bioengineering Institute, Nanjing, China).

### 3.6. Quantitative Real-Time PCR

After completion of the pain tests on day 21, the rats were anesthetized with pentobarbital sodium (50 mg/kg), and the ipsilateral lumbar spinal cord dorsal horn (L5-L6) and the corresponding DRG sections were collected after the rats were perfused transcardially with 0.9% saline. The samples were stored at -80°C until further use.

Total RNA was separately isolated from the spinal cord and DRG tissues using the TRIzol Reagent (Invitrogen, USA) according to the manufacturer's instructions. SuperScript II Reverse Transcriptase (Invitrogen, USA) was used in the present study to synthesize cDNA from total RNA. Then, quantitative real-time PCR was performed using the SYBR Green qPCR Master Mix (Thermo Fisher, USA) in an ABI ViiA 7 Dx instrument (Applied Biosystems, USA). The primer sequences are displayed in Table 1. The qRT-PCR was performed in duplicate, and the expression of each gene was quantified using the 2^−*ΔΔ*Ct^ method, and results were normalized to those of GAPDH.

### 3.7. Assay of SIRT1 Activity

SIRT1 activity was measured according to the previous report [17]. Briefly, the spinal cord and DRG lysate were prepared using GENMED lysis buffer. Then, the substrate (5 *μ*L), buffer solution (55 *μ*L), and replenisher (20 *μ*L) were transferred to a 96-well plate and mixed. Afterwards, the mixture was incubated at 30°C for 1 h, and the reaction was terminated by adding a stop solution (10 *μ*L) followed by an enzymolysis liquid (10 *μ*L). The fluorescence intensity was measured with a microplate reader at 405 nm.

### 3.8. Statistical Analysis

Data were expressed as the mean ± standard deviation (SD). The data from pain behavioral tests at different time points were analyzed by two-way repeated-measures ANOVA followed by a Bonferroni test for post hoc analysis (GraphPad Software, CA, USA). The data from ELISA and mRNA were analyzed by one-way ANOVA followed by a Bonferroni test for post hoc analysis. *P* < 0.05 was considered statistically significant.

## 4. Results

### 4.1. Preliminary Chemical Characterization of GBP

**Figure 2:**
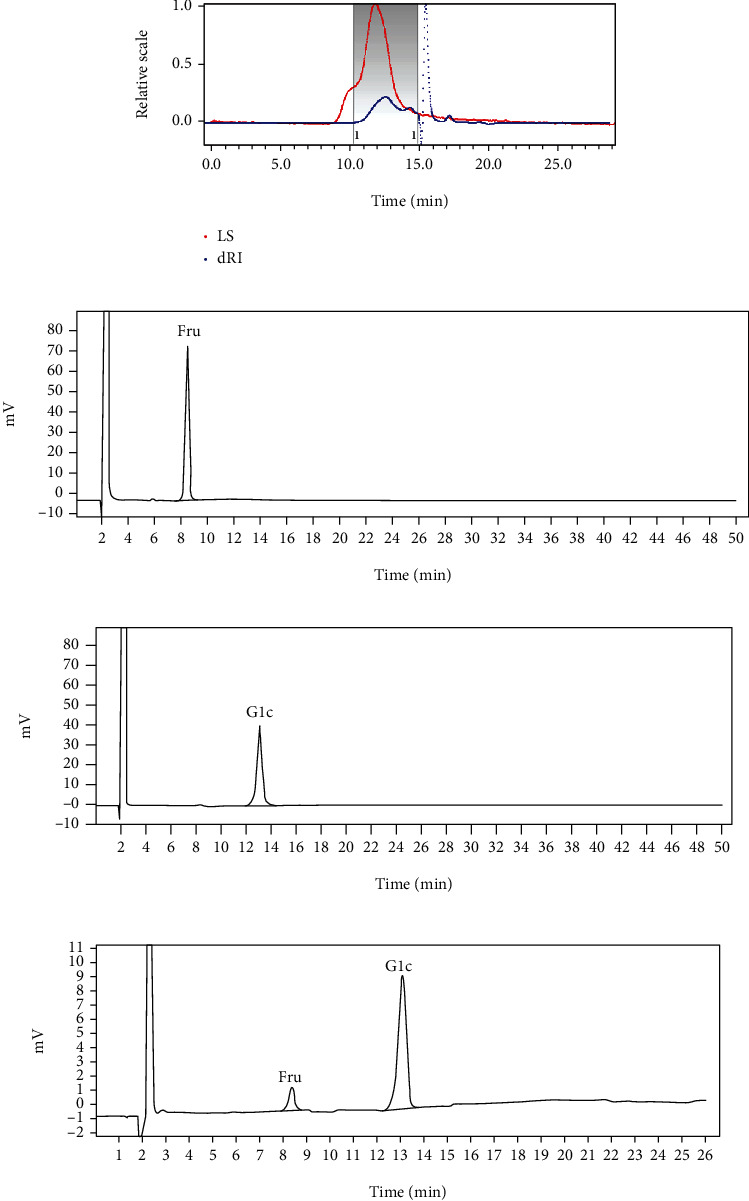
GPC-MALLS-RI chromatography of GBP (a). HPLC-RI analysis of fructose (b), glucose (c), and GBP hydrolyzates (d).

The GPC-MALLS-RI was used to measure the molecular weight and homogeneity of GBP. As shown in Table 2 and Figure 2(a), the Mw and polydispersity of LBP were 53.43 kDa and 1.26, respectively. Besides, the carbohydrate content of GBP was 94.01 ± 0.21%. A HPLC-RI analytical method was performed to analyze the monosaccharide composition of GBP. Table 2 and Figures 2(b)–2(d) indicated that GBP was composed of fructose and glucose in a molar ratio of 1 : 8.70.

### 4.2. Effects of GBP on Pain in Normal Rats

As shown in Figures 1(b) and 1(c), GBP administration (20 mg/kg, once a day for 21 consecutive days) did not impact the paw withdrawal latency and the paw withdrawal threshold in normal rats.

### 4.3. Effects of GBP on Vin-Induced Neuropathic Pain

**Figure 3:**
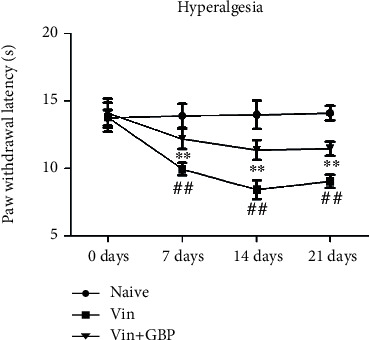
*G. elata* polysaccharide (GBP) treatment relieves pain hypersensitivity in vincristine-induced neuropathic pain rats. Paw withdrawal latency in different groups. Data were expressed as the mean ± SD (*n* = 8 for each group). ^#^*P* < 0.01: vs. naive group; ^∗∗^*P* < 0.01: vs. Vin group. Two-way ANOVA followed by Bonferroni's post hoc analysis.

**Figure 4:**
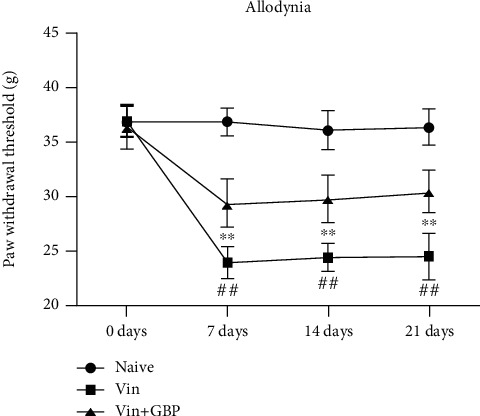
*G. elata* polysaccharide (GBP) treatment relieves pain hypersensitivity in vincristine-induced neuropathic pain rats. Paw withdrawal threshold in different groups. Data were expressed as the mean ± SD (*n* = 8 for each group). ^#^*P* < 0.01: vs. naive group; ^∗∗^*P* < 0.01: vs. Vin group. Two-way ANOVA followed by Bonferroni's post hoc analysis.

As shown in Figures 3 and 4, a significant decrease of the paw withdrawal latency and the paw withdrawal threshold was observed in the Vin group as compared with the naive group (*P* < 0.01), indicating that Vin (0.1 mg/kg) injection caused thermal hyperalgesia and mechanical allodynia which persisted for the entire observation period. However, these abnormal changes were reversed by GBP administration for 21 consecutive days (*P* < 0.01). These results indicated that GBP treatment alleviated Vin-induced neuropathic pain.

### 4.4. Effects of GBP on Sciatic Nerve Histopathological Changes

**Figure 5:**
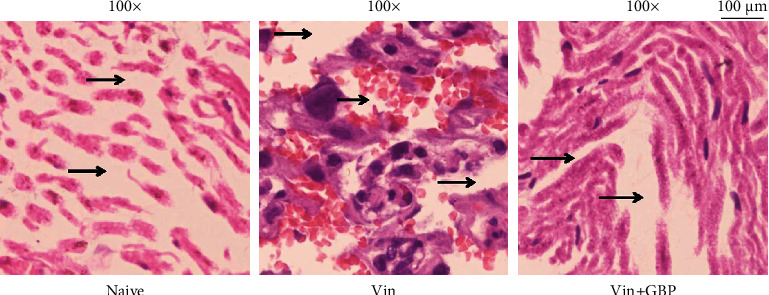
Effects of *G. elata* polysaccharides (GBP) on histopathological changes of the sciatic nerve in Vin-induced neuropathic pain (C). The black arrows indicated neuron gaps and fiber arrangement. Sciatic nerve tissues were stained using the H&E method and observed under light microscopy (100x). Scale bar = 100 *μ*m.

In the present study, the sciatic nerves were stained with hematoxylin and eosin to assess whether GBP alleviated the histopathological alterations in the sciatic nerve, further revealing the neuroprotective action of GBP in the Vin-induced neuropathic pain model. As shown in Figure 5, Vin injection caused histopathological changes such as the development of neuron gaps, axonal selling, and disorientation of the nerve fibers in the Vin group, in comparison with the naive group. However, GBP treatment alleviated the Vin-induced histopathological alterations.

### 4.5. Effects of GBP Administration on the Levels of Proinflammatory Cytokines in the Sciatic Nerve, Spinal Cord, and DRG

**Figure 6:**
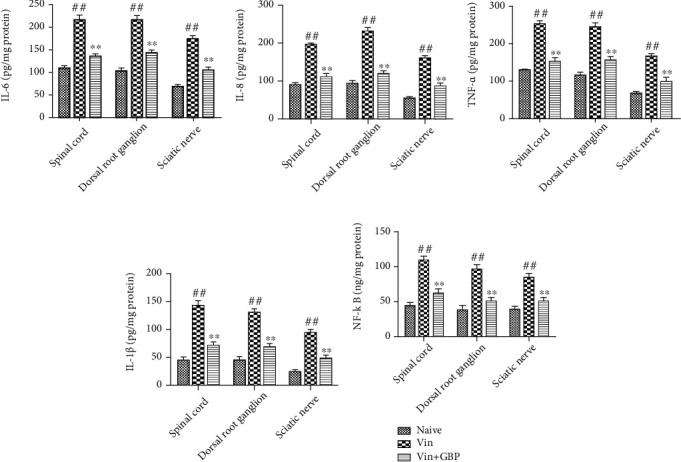
Effects of *G. elata* polysaccharide (GBP) administration on the levels of proinflammatory cytokines in Vin-induced neuropathic pain rats. After completion of the pain behavioral tests on day 21, levels of IL-6 (a), IL-8 (b), TNF-*α* (c), IL-1*β* (d), and NF-*κ*B (e) in the sciatic nerve, spinal cord, and dorsal root ganglion (DRG) were measured. Data were expressed as the mean ± SD (*n* = 8 for each group). ^#^*P* < 0.01: vs. naive group; ^∗∗^*P* < 0.01: vs. Vin group. One-way ANOVA followed by Bonferroni's post hoc analysis.

As shown in Figures 6(a)–6(d), compared with the naive group, IL-6, IL-8, TNF-*α*, and IL-1*β* levels were significantly increased in the sciatic nerve, spinal cord, and DRG of the Vin group (*P* < 0.01). However, oral administration of GBP decreased the Vin-induced increase of IL-6, IL-8, TNF-*α*, and IL-1*β* (*P* < 0.01).

### 4.6. Effects of GBP Administration on NF-*κ*B Activation in the Sciatic Nerve, Spinal Cord, and DRG

As shown in Figure 6(e), compared with the naive group, NF-*κ*B activation was significantly increased in the spinal cord and DRG of rats in the Vin group (*P* < 0.01). However, oral administration of GBP decreased the Vin-induced increase of NF-*κ*B level in the spinal cord and DRG (*P* < 0.01).

### 4.7. Effects of GBP Administration on the mRNA Expression of Proinflammatory Cytokines in the Sciatic Nerve, Spinal Cord, and DRG

**Figure 7:**
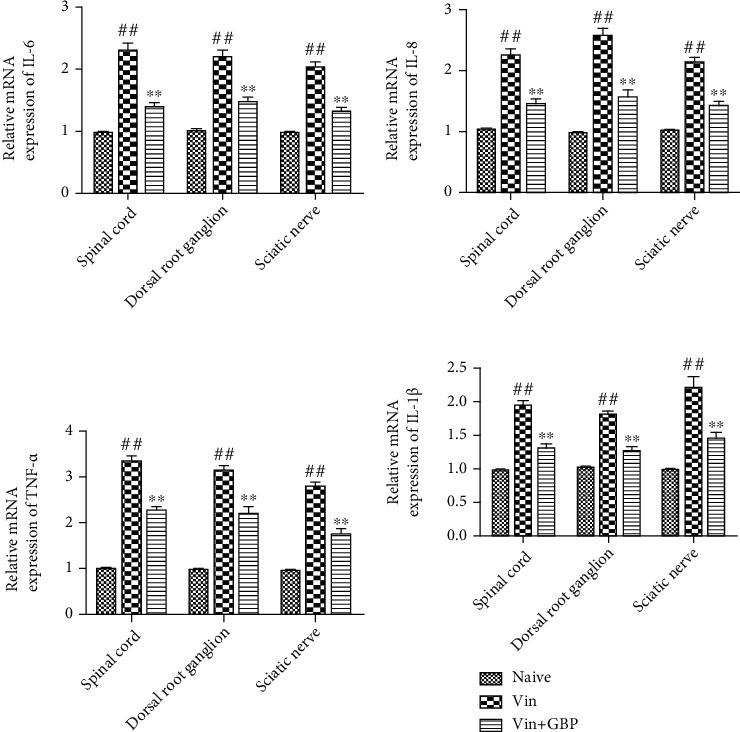
Effects of *G. elata* polysaccharide (GBP) administration on the mRNA expression of proinflammatory cytokines in the sciatic nerve, spinal cord, and dorsal root ganglion (DRG). After completion of the pain behavioral tests on day 21, sciatic nerve, spinal cord, and dorsal root ganglion (DRG) were collected. mRNA expression of IL-6 (a), IL-8 (b), TNF-*α* (c), and IL-1*β* (d) was measured. Data were expressed as the mean ± SD (*n* = 6 for each group). ^#^*P* < 0.01: vs. naive group; ^∗∗^*P* < 0.01: vs Vin group. One-way ANOVA followed by Bonferroni's post hoc analysis.

Similarly, the mRNA expression data of IL-6, IL-8, TNF-*α*, and IL-1*β* were consistent with the ELISA results (Figure 7). Compared with the naive group, IL-6, IL-8, TNF-*α*, and IL-1*β* mRNA expressions were significantly increased in the sciatic nerve, spinal cord, and DRG of rats in the Vin group (*P* < 0.01). However, oral administration of GBP downregulated the upregulation of IL-6, IL-8, TNF-*α*, and IL-1*β* mRNA expressions induced by Vin (*P* < 0.01).

### 4.8. GBP Alleviated Vin-Induced Neuropathic Pain Partly via Activating SIRT1

**Figure 8:**
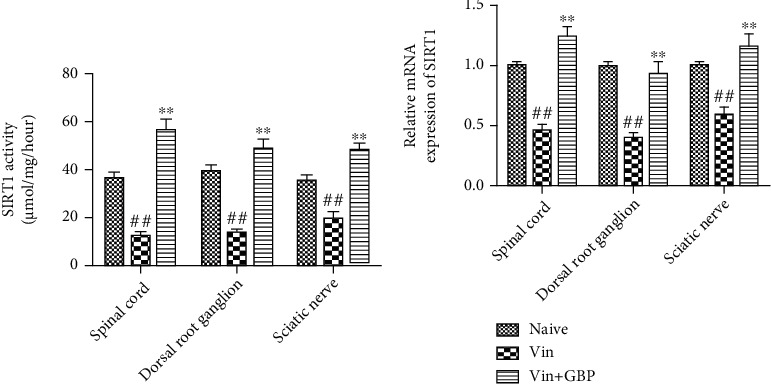
*G. elata* polysaccharides (GBP) alleviated Vin-induced neuropathic pain via activating the SIRT1 in rats. After completion of the pain behavioral tests on day 21, the sciatic nerve, spinal cord, and dorsal root ganglion (DRG) were collected. SIRT1 activity (a) and mRNA expression of SIRT1 (b) were measured. Data were expressed as the mean ± SD (*n* = 6 for each group). ^#^*P* < 0.01: vs. naive group; ^∗∗^*P* < 0.01: vs. Vin group. One-way ANOVA followed by Bonferroni's post hoc analysis.

As shown in Figure 8, compared with the naive group, SIRT1 activity and expression were significantly decreased in the sciatic nerve, spinal cord, and DRG of rats in the Vin group (*P* < 0.01). However, oral administration of GBP alleviated the Vin-induced SIRT1 changes in the sciatic nerve, spinal cord, and DRG, as evidenced by the improved activity and expression of SIRT1 compared with the Vin group (*P* < 0.01).

## 5. Discussion

Based on the recent reports, lots of the clinically available drugs are simply aimed at the relief of the pain symptom, and there is no powerful drug that has been approved for the prevention and treatment of neuropathic pain [18]. As a traditional Chinese medicine, *G. elata* is known for its anti-inflammatory and analgesic effects and has been used clinically for the treatment of limb numbness, epilepsy, dizziness, and headaches. GBP is the primary active constituent of *G. elata* root that exerted several pharmacological activities. However, there is little report about the analgesic effect of GBP in the chemotherapy-induced neuropathy model. In accordance with previous studies [19, 20], our results indicated that Vin increased thermal and mechanical sensitivity, and this was accompanied by an increase of proinflammatory cytokine levels and downregulation of SIRT1. Besides, the antinociceptive effect of GBP against Vin-induced neuropathic pain in rats was investigated for the first time, and the results showed that GBP decreased proinflammatory cytokine levels and activated SIRT1 expression in the spinal cord and DRG. These biochemical alterations were verified by the histopathological assessment that observed significant sciatic nerve damage in the Vin group. And these abnormal alterations, including histopathological and biochemical alternations, were restored by GBP suggesting its efficacy to alleviate Vin-induced neuropathic pain.

Neuropathic pain is considered a neuroimmune disorder. It has been reported that proinflammatory cytokines, including IL-6, IL-8, IL-1*β*, and TNF-*α*, are released after the activation of immune-like glial cells in the spinal cord and DRG [21]. These proinflammatory factors could also activate the nearby neurons or glia to accelerate neuroinflammation through nerve damage-evoked persistent pain [22]. Recently, neuroinflammation has been focused on as an important regulator in the pathology of neuropathic pain [23]. Besides, the overexpression levels of proinflammatory factors were observed in the spinal cord of neuropathic pain animal models, and the administration of anti-inflammatory agents inhibited inflammatory responses and alleviated neuropathic pain [24, 25]. NF-*κ*B is a vital transcription factor, plays an important role in regulating the gene expression of inflammatory factors, and is involved in the initiation, persistence, and severity of neuropathic pain following nerve impairment [26]. Thus, suppression of inflammatory responses is considered to be a novel approach for the attenuation of neuropathic pain [27, 28]. In the present study, GBP treatment obviously decreased the NF-*κ*B level in the spinal cord and DRG which consequently suppressed IL-6, IL-8, IL-1*β*, and TNF-*α* release. These results showed that GBP could inhibit Vin-induced neuroinflammation.

Sirtuin1 (SIRT1) is a nicotinamide adenine dinucleotide-dependent protein lysine deacetylase, which has traditionally been related to lifespan extension, inflammation, and aging in mammals [29, 30]. Several reports revealed that activating SIRT1 plays an important role in alleviating tissue damage in various animal models. It has been reported that SIRT1 is involved in molecular pathways associated with nerve damage and neuropathic pain [31, 32]. Therefore, SIRT1 has also been indicated to be a potential target for the prevention and treatment of neuropathic pain [6, 33, 34]. Our findings indicated that GBP administration improved SIRT1 activity and expression in the spinal cord and DRG, and this improvement was consistent with the pain behavioral alterations, implying the analgesic effect of GBP on Vin-induced neuropathic pain via the regulation of SIRT1.

In conclusion, our investigation provides evidence that GBP could relieve neuropathic pain, possibly through inhibiting neuroinflammation. The present research also suggests that GBP may be a promising therapeutic agent for the management and alleviation of neuropathic pain.

## Figures and Tables

**Table 1:** Sequences of primers used quantitative real-time PCR.

Gene	Forward primer	Reverse primer
IL-6	5′-TGGTGATAAATCCCGATGAAG-3′	5′-GGCACTGAAACTCCTGGTCT-3′
IL-8	5′-GGTAAAGTCCGTAAGTCGTAGTAC-3′	5′-AGTTAACGTGGAGGTACCGTA-3′
TNF-*α*	5′-CCACGCTCTTCTGTCTACTG-3′	5′-GCTACGGGCTTGTCACTC-3′
SIRT1	5′-ATTTATGCTCGCCTTGCTGT-3′	5′-GATCCTTTGGATTCCTGCAA-3′
IL-1*β*	5′-GCCGATGGTCCCAATTACAT-3′	5′-ACAAGACCTGCCGGAAGCT-3′
GAPDH	5′-CAACTTTGGCATTGTGGAAGG-3′	5′-ACACATTGGGGGTAGGAACAC-3′

**Table 2:** The chemical analysis of polysaccharides from *G. elata* (GBP).

Sample	GBP
Mw (kDa)	53.43 ± 0.83
Polydispersity (Mw/Mn)	1.26
Carbohydrate content (%)	94.01 ± 0.21
Monosaccharide composition	Fructose : glucose = 1 : 8.70

## Data Availability

The datasets used during the current study are available from the corresponding author on reasonable request.

## References

[1] Nascimento O. J., Pessoa B. L., Orsini M. (2016). Neuropathic pain treatment: still a challenge. *Neurology International*.

[2] Wang Y., Cao S. E., Tian J., Liu G., Zhang X., Li P. (2013). Retracted article: auraptenol attenuates vincristine-induced mechanical hyperalgesia through serotonin 5-HT_1A_ receptors. *Scientific Reports*.

[3] Brewer J. R., Morrison G., Dolan M. E., Fleming G. F. (2016). Chemotherapy-induced peripheral neuropathy: current status and progress. *Gynecologic Oncology*.

[4] Moalem G., Tracey D. J. (2006). Immune and inflammatory mechanisms in neuropathic pain. *Brain Research Reviews*.

[5] Ledeboer A., Jekich B. M., Sloane E. M. (2007). Intrathecal interleukin-10 gene therapy attenuates paclitaxel-induced mechanical allodynia and proinflammatory cytokine expression in dorsal root ganglia in rats. *Brain, Behavior, and Immunity*.

[6] Zhang Z., Ding X., Zhou Z. (2019). Sirtuin 1 alleviates diabetic neuropathic pain by regulating synaptic plasticity of spinal dorsal horn neurons. *Pain*.

[7] Chu Y., Ge W., Wang X. (2019). MicroRNA-448 modulates the progression of neuropathic pain by targeting sirtuin 1. *Experimental and Therapeutic Medicine*.

[8] Ojemann L. M., Nelson W. L., Shin D. S., Rowe A. O., Buchanan R. A. (2006). Tian ma, an ancient Chinese herb, offers new options for the treatment of epilepsy and other conditions. *Epilepsy & behavior: E&B*.

[9] Ahn E. K., Jeon H. J., Lim E. J., Jung H. J., Park E. H. (2007). Anti-inflammatory and anti-angiogenic activities of _Gastrodia elata_ Blume. *Journal of Ethnopharmacology*.

[10] Wang L., Zhang J., Hong Y., Feng Y., Chen M., Wang Y. (2013). Phytochemical and pharmacological review of da chuanxiong formula: a famous herb pair composed of chuanxiong rhizoma and gastrodiae rhizoma for headache. *Evidence-based Complementary and Alternative Medicine: Ecam*.

[11] Zhu H., Liu C., Hou J. (2019). Gastrodia elata Blume polysaccharides: a review of their acquisition, analysis, modification, and pharmacological activities. *Molecules*.

[12] Lee O. H., Kim K. I., Han C. K., Kim Y. C., Hong H. D. (2012). Effects of acidic polysaccharides from Gastrodia rhizome on systolic blood pressure and serum lipid concentrations in spontaneously hypertensive rats fed a high-fat diet. *International Journal of Molecular Sciences*.

[13] Zhou B., Tan J., Zhang C., Wu Y. (2018). Neuroprotective effect of polysaccharides from Gastrodia elata blume against corticosterone-induced apoptosis in PC12 cells via inhibition of the endoplasmic reticulum stress-mediated pathway. *Molecular Medicine Reports*.

[14] Chen J., Tian S., Shu X., Du H., Li N., Wang J. (2016). Extraction, characterization and immunological activity of polysaccharides from Rhizoma gastrodiae. *International journal of molecular sciences*.

[15] Gautam M., Ramanathan M. (2019). Saponins of Tribulus terrestris attenuated neuropathic pain induced with vincristine through central and peripheral mechanism. *Inflammopharmacology*.

[16] Kim K. J., Lee O. H., Han C. K., Kim Y. C., Hong H. D. (2012). Acidic polysaccharide extracts from Gastrodia rhizomes suppress the atherosclerosis risk index through inhibition of the serum cholesterol composition in Sprague Dawley rats fed a high-fat diet. *International Journal of Molecular Sciences*.

[17] Du L. L., Xie J. Z., Cheng X. S. (2014). Activation of sirtuin 1 attenuates cerebral ventricular streptozotocin-induced tau hyperphosphorylation and cognitive injuries in rat hippocampi. *Age*.

[18] Ma J., Kavelaars A., Dougherty P. M., Heijnen C. J. (2018). Beyond symptomatic relief for chemotherapy-induced peripheral neuropathy: targeting the source. *Cancer*.

[19] Kiguchi N., Maeda T., Kobayashi Y., Saika F., Kishioka S. (2009). Chapter 14 involvement of inflammatory mediators in neuropathic pain caused by vincristine. *International Review of Neurobiology*.

[20] Jaggi A. S., Singh N. (2012). Mechanisms in cancer-chemotherapeutic drugs-induced peripheral neuropathy. *Toxicology*.

[21] Mika J., Zychowska M., Popiolek-Barczyk K., Rojewska E., Przewlocka B. (2013). Importance of glial activation in neuropathic pain. *European Journal of Pharmacology*.

[22] Siemian J. N., LaMacchia Z. M., Spreuer V. (2018). The imidazoline I_2_ receptor agonist 2-BFI attenuates hypersensitivity and spinal neuroinflammation in a rat model of neuropathic pain. *Biochemical Pharmacology*.

[23] Sommer C., Leinders M., Üçeyler N. (2018). Inflammation in the pathophysiology of neuropathic pain. *Pain*.

[24] Jiang K., Shi J., Shi J. (2019). Morin alleviates vincristine-induced neuropathic pain via nerve protective effect and inhibition of NF-*κ*B pathway in rats. *Cellular and Molecular Neurobiology*.

[25] Alonso-Castro A. J., Arana-Argáez V., Yáñez-Barrientos E. (2020). Antinociceptive and anti-inflammatory effects of Cuphea aequipetala Cav (Lythraceae). *Inflammopharmacology*.

[26] Sakaue G., Shimaoka M., Fukuoka T. (2001). NF-kappa B decoy suppresses cytokine expression and thermal hyperalgesia in a rat neuropathic pain model. *Neuroreport*.

[27] Liu M., Liao K., Yu C., Li X., Liu S., Yang S. (2014). Puerarin alleviates neuropathic pain by inhibiting neuroinflammation in spinal cord. *Mediators of Inflammation*.

[28] Li Y., Chen C., Li S., Jiang C. (2019). Ginsenoside Rf relieves mechanical hypersensitivity, depression-like behavior, and inflammatory reactions in chronic constriction injury rats. *Phytotherapy research: PTR*.

[29] Brooks C. L., Gu W. (2009). How does SIRT1 affect metabolism, senescence and cancer?. *Nature Reviews Cancer*.

[30] Chang H. C., Guarente L. (2014). SIRT1 and other sirtuins in metabolism. *Trends in Endocrinology and Metabolism: TEM*.

[31] Araki T., Sasaki Y., Milbrandt J. (2004). Increased nuclear NAD biosynthesis and SIRT1 activation prevent axonal degeneration. *Science*.

[32] Renthal W., Kumar A., Xiao G. (2009). Genome-wide analysis of chromatin regulation by cocaine reveals a role for sirtuins. *Neuron*.

[33] Shao H., Xue Q., Zhang F. (2014). Spinal SIRT1 activation attenuates neuropathic pain in mice. *PLoS One*.

[34] Lv C., Hu H. Y., Zhao L., Zheng H., Luo X. Z., Zhang J. (2015). Intrathecal SRT1720, a SIRT1 agonist, exerts anti-hyperalgesic and anti-inflammatory effects on chronic constriction injury-induced neuropathic pain in rats. *International Journal of Clinical and Experimental Medicine*.

